# Association of smoking status with non-small cell lung cancer patients harboring uncommon epidermal growth factor receptor mutation

**DOI:** 10.3389/fimmu.2022.1011092

**Published:** 2022-10-20

**Authors:** How-Wen Ko, Shian-Sen Shie, Chih-Wei Wang, Chi-Tsun Chiu, Chih-Liang Wang, Tsung-Ying Yang, Shou-Chu Chou, Chien-Ying Liu, Chih-Hsi Scott Kuo, Yu-Ching Lin, Li-Fu Li, Cheng-Ta Yang, Chin-Chou Wang

**Affiliations:** ^1^ Division of Thoracic Oncology, Department of Thoracic Medicine, Linkou Chang Gung Memorial Hospital, College of Medicine, Chang Gung University, Taoyuan, Taiwan; ^2^ Department of Internal Medicine, Linkou Chang Gung Memorial Hospital, College of Medicine, Chang Gung University, Taoyuan, Taiwan; ^3^ Department of Pathology, Linkou Chang Gung Memorial Hospital, College of Medicine, Chang Gung University, Taoyuan, Taiwan; ^4^ Institute of European and American Studies, Academia Sinica, Taipei, Taiwan; ^5^ Division of Chest Medicine, Department of Internal Medicine, Taichung Veterans General Hospital, Taichung, Taiwan; ^6^ Department of Family Medicine, Shuang Ho Hospital, Taipei Medical University, New Taipei, Taiwan; ^7^ Department of Thoracic Medicine, Taipei Chang Gung Memorial Hospital, Taipei, Taiwan; ^8^ Department of Pulmonary and Critical Care Medicine, Chiayi Chang Gung Memorial Hospital, College of Medicine, Chang Gung University, Puzi, Taiwan; ^9^ Division of Pulmonary and Critical Care Medicine, Department of Internal Medicine, Keelung Chang Gung Memorial Hospital, College of Medicine, Chang Gung University, Keelung, Taiwan; ^10^ Department of Thoracic Medicine, Taoyuan Chang Gung Memorial Hospital, Taoyuan, Taiwan; ^11^ Department of Respiratory Therapy, College of Medicine, Chang Gung University, Taoyuan, Taiwan; ^12^ Divisions of Pulmonary & Critical Care Medicine, Department of Internal Medicine, Kaohsiung Chang Gung Memorial Hospital, College of Medicine, Chang Gung University, Kaohsiung, Taiwan

**Keywords:** smoking, epidermal growth factor receptor (EGFR), non-small cell lung cancer (NSCLC), uncommon *EGFR* mutation, complex *EGFR* mutation

## Abstract

**Introduction:**

Uncommon epidermal growth factor receptor (EGFR) mutations include single and complex mutations. However, the association of the smoking status of patients with uncommon and complex *EGFR* mutations remains unclear.

**Methods:**

This retrospective study evaluates the spectrum of uncommon *EGFR* mutations and investigates the influence of smoking status on the frequency of various uncommon *EGFR* mutations using a multi-institutional medical database.

**Results:**

Between 2010 and 2019, 5,608 non-small cell lung cancer (NSCLC) patients were analyzed. *EGFR* mutations were detected in 3,155 (56.3%) patients. Among the 399 (12.6%) patients with uncommon mutations, 198 had single uncommon and 201 complex mutations, including 87 exon 20 insertions, 79 *de novo* T790M, 70 complex common, and 52 complex uncommon mutations. For comparison, we also included 402 patients with common *EGFR* mutations. The percentage of ever-smokers was significantly higher in patients with uncommon *EGFR* mutations than in patients with common *EGFR* mutations (25.8% vs. 17.4%, *p* = 0.005). Furthermore, the percentage of ever-smokers was higher in those with a complex mutation than in those with a single uncommon mutation (30.3% vs. 21.2%, *p* = 0.040). Among patients carrying uncommon *EGFR* mutations, ever-smokers had significantly more complex uncommon *EGFR* mutations than never-smokers (22.3% vs. 9.8%, *p* = 0.002). Among patients carrying G719X, L861Q, and S768I, ever-smokers tended to have complex *EGFR* mutations more frequently than never-smokers (64.7% vs. 28.7%, 50.0% vs. 18.7%, 88.9% vs. 81.2%, respectively).

**Conclusions:**

Our study demonstrates not only a comprehensive spectrum of uncommon *EGFR* mutations, but also a positive relationship between smoking status and uncommon *EGFR* mutation frequency, especially complex uncommon *EGFR* mutations. The results suggest that smoking contributes to the development of complex *EGFR* mutations.

## Introduction

Lung cancer has been the leading cause of cancer-related deaths for several years worldwide, and non-small cell lung cancer (NSCLC) accounts for 85–90% of all cases (1). Smoking is the most important risk factor that causes lung cancer (2). Studies have shown that the pathogenesis and clinical manifestations of ever- and never-smokers are different, and it has also been verified that smoking is associated with poor therapeutic outcomes and decreased survival (2).

Epidermal growth factor receptor (EGFR) mutations are the most commonly detected and targetable driver mutations in NSCLC (3). Approximately 50% of Asian and 8–16% of non-Asian patients with NSCLC harbor *EGFR* mutation (4). Exon 19 deletions and *L858R* substitutions in exon 21 account for approximately 85–90% of all *EGFR* mutations, which are known to be common mutations and are sensitive to *EGFR*-tyrosine kinase inhibitors (TKIs). Patients with advanced NSCLC and common *EGFR* mutations have a higher response rate and longer progression-free survival (PFS) when treated with first-line *EGFR*-TKIs than with platinum-based chemotherapy (5). Further randomized clinical trials have validated the overall survival (OS) advantage of second- and third-generation over first-*EGFR*-TKIs (6, 7). Hence, routine testing for *EGFR* mutation status has become a standard-of-care recommendation for advanced NSCLC patients, especially those with lung adenocarcinoma histology (8, 9).

*EGFR* mutations, other than exon 19 deletions and *L858R* mutations, are uncommon. These uncommon mutations are highly heterogeneous and demonstrate variable responses to *EGFR*-TKIs, which remain poorly characterized. The most frequently found uncommon mutations include G719X, L861Q, S768I, exon 20 insertions (Ex20ins), and *de novo* T790M (10). NSCLC carrying Ex20ins and *de novo* T790M are resistant to both first- and second-generation *EGFR*-TKI therapies, whereas other uncommon mutations are usually sensitive (11). Information regarding the activity of *EGFR*-TKIs against uncommon *EGFR* mutations are limited (11, 12). Chiu et al. (13) reported that first-generation *EGFR*-TKIs were less effective in patients with the G719X, L861Q, and S768I mutations than in those with common mutations. Recently, Yang et al. conducted a *post-hoc* analysis of 1023 cases and showed that a second-generation *EGFR*-TKI, afatinib, was highly effective in patients with certain types of uncommon mutations (14).

Uncommon *EGFR* mutations can occur alone or coexist with either common or other uncommon *EGFR* mutations, including complex or compound mutations. The incidence of complex *EGFR* mutations varies among different study populations and detection methods, ranging from 4% to 26% of all *EGFR* mutations (15). Data regarding the effect of *EGFR*-TKIs on complex *EGFR* mutations are even fewer, and the results based on a relatively small number of cases were highly heterogeneous (15). Generally, the result in patients carrying complex mutations is similar to that in patients with single uncommon mutations and is less favorable than that in patients with common mutations (15, 16). Nevertheless, the characteristics of NSCLC patients with complex *EGFR* mutations are not fully understood.

Previous studies have identified several clinical features related to the prevalence of *EGFR* mutations in NSCLC such as female sex, Asian ethnicity, lung adenocarcinoma, and never-smoking status (17–19). *EGFR* mutations occur more frequently in non-smokers than in smokers. In these earlier studies, the majority of the detected *EGFR* mutations were common (20, 21). Despite the strong association between the prevalence of *EGFR* mutations and never-smoking status, *EGFR* mutations can still be detected in smokers. Smoking is known to increase tumor mutation burden (22). Moreover, advanced detection techniques have broadened *EGFR* mutation spectrum, and uncommon or complex *EGFR* mutations have been identified (10, 23). It remains unclear whether smoking status correlates with the pattern of uncommon *EGFR* mutations and affects the development of complex *EGFR* mutations.

In the present study, we use a multi-institutional database from 2010 to 2019 to examine the uncommon *EGFR* mutation spectrum. We compare the frequencies of uncommon *EGFR* mutation subtypes between ever- and never-smokers, while investigating the smoking status of patients harboring uncommon *EGFR* genotypes.

## Materials and methods

### Patient population

**Figure 1:**
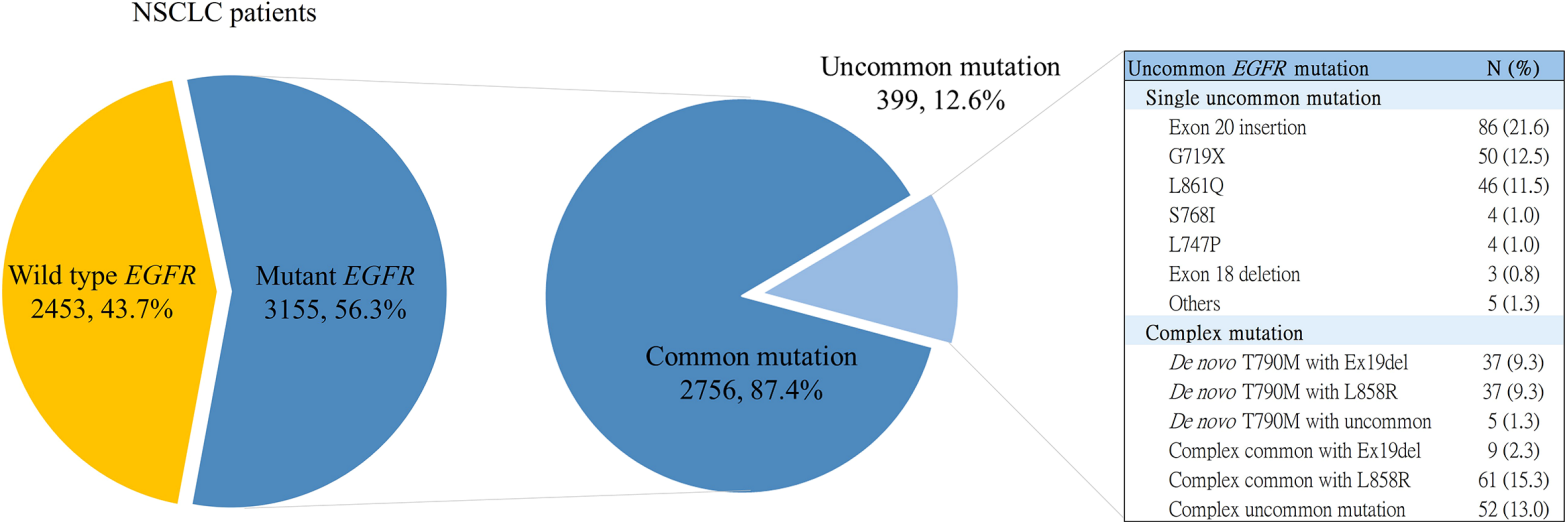
NSCLC patients receiving *EGFR* mutation testing and distribution of uncommon *EGFR* mutation. *EGFR*, epidermal growth factor receptor; Ex19del, exon 19 deletion; NSCLC, non-small cell lung cancer.

This retrospective cohort study accesses patient data from the Chang Gung Research Database, a multi-institutional electronic medical record collection system in Taiwan (24). The screening criteria were as follows (1): NSCLC patients who were treated at any one of the institutions of Chang Gung Memorial Hospitals, including Linkou, Kaohsiung Medical Centers, Taipei, Taoyuan, Keelung, and Chiayi branches; and (2) patients with histologically or cytologically confirmed NSCLC who underwent *EGFR* mutation analysis at Chang Gung Memorial Hospital, Linkou Medical Center between 2010 and 2019. A total of 5,608 patients were screened. Of the 3,155 patients carrying *EGFR* mutations, 399 had uncommon *EGFR* mutations (**Figure 1**). Clinical data of patients with uncommon *EGFR* mutations were recorded, including age at diagnosis, sex, smoking status, Eastern Cooperative Oncology Group (ECOG) performance status, histology type, tumor stage (according to the 8th edition of the American Joint Committee on Cancer staging system), and *EGFR* mutation type. Two thousand, seven hundred and fifty-six patients had common *EGFR* mutations. We filtered patients’ information using the following criteria: (1) NSCLC patients who were treated between 2016 and 2017; (2) positive common *EGFR* mutation; and (3) who were at advanced or metastatic stage and received Taiwan’s National Health Insurance reimbursed first-line treatment with *EGFR*-TKIs. Thus, 402 patients were enrolled for comparison. This study was approved by the Institutional Review Board of Chang Gung Memorial Hospital (No. 202200840B0).

### EGFR mutation testing

*EGFR* mutation status was detected using polymerase chain reaction (PCR)-direct sequencing or mutant type-specific sensitive methods based on the tumor purity of tissue samples (25). Mutant-type-specific sensitive methods include the Scorpions amplification-refractory mutation system (ARMS) (Therascreen EGFR RGQ PCR Kit, Qiagen) and competitive allele-specific TaqMan PCR (Life Technologies) (25, 26). For PCR-direct sequencing, DNA was extracted from tumor specimens for *EGFR* mutation analysis, as described previously (25, 27). For mutant type-specific sensitive methods, DNA extraction and analysis were performed using commercial kits in accordance with the manufacturer’s instructions.

Exon 19 deletions (Ex19del) and L858R are common *EGFR* mutations. All *EGFR* mutations other than the common ones were uncommon, including G719X, L861Q, S768I, exon 20 insertion (Ex20ins), and *de novo* T790M. Two or more *EGFR* mutations within the same tumor tissue were defined as complex *EGFR* mutations, encompassing *de novo* T790M, complex common, and complex uncommon *EGFR* mutations. *De novo* T790M mutations are primary T790M mutations that are accompanied by common or uncommon *EGFR* mutations. Complex common *EGFR* mutations are common mutations that coexist with one or more uncommon mutations. Complex uncommon *EGFR* mutations are two or more distinct uncommon mutations within the same tumor tissue. The distribution of uncommon *EGFR* mutations is shown in **Figure 1**.

### Statistical analysis

Fisher's exact test and Pearson’s chi-squared test were used to compare categorical variables, including clinical factors, the type and frequency of *EGFR* mutations, and smoking status. A logistic regression was employed to calculate the odds ratios (ORs) and 95% confidence intervals (CIs) for univariate and multivariate analyses to assess the independent association between different variables and presence of common versus uncommon *EGFR* mutations. For evaluation of the association of smoking status with the type and frequency of uncommon *EGFR* mutations, patients with NSCLC carrying uncommon *EGFR* mutations were categorized as never-smokers or ever-smokers based on their self-reported smoking status. Never-smokers were defined as those who smoked fewer than 100 cigarettes during their lifetime. Other patients were defined as ever-smokers, including both current and former smokers. We evaluated the correlation between smoking status and the frequency of uncommon *EGFR* mutations, and compared the mutation types between ever- and never-smokers with NSCLC histology carrying uncommon *EGFR* mutations. A two-sided *p* value less than 0.05 was considered statistically significant. Statistical analyses were performed using GraphPad Prism (version 5.02, GraphPad Software, La Jolla, CA, USA) and R software (version 4.1.2).

## Results

### Patient characteristics

A total of 399 patients with NSCLC harboring uncommon *EGFR* mutations were enrolled in the study. The baseline characteristics of the patients are shown in **Table 1**. The median age during diagnosis was 65 years (range:32–94 years). Most patients were female (61.7%), never-smokers (74.2%), had an ECOG performance status of 0–1 (80.7%), were diagnosed with adenocarcinoma histology (93.7%), and had advanced- or metastatic- staged diseases at baseline (89.7%).

Another set of 402 NSCLC patients with common *EGFR* mutations who were diagnosed between 2016 and 2017 were included for comparison. All patients had stage IIIB/IIIC or stage IV cancer. The demographic data of the 402 patients were similar to those of the 399 patients, except for smoking status (**Table 1**). The percentage of ever-smokers among patients with uncommon *EGFR* mutations was significantly higher than that among patients with common *EGFR* mutations (25.8% vs. 17.4%, *p* = 0.005). Furthermore, we performed logistic regression to assess the independent association between different variables and presence of common versus uncommon *EGFR* mutations. We found that ever smoking history was significantly correlated with uncommon *EGFR* mutation status (**Table 2**).

**Table 1:** Demographic data of NSCLC patients harboring common and uncommon *EGFR* mutations.

Characteristic, N (%)	Common mutation	Uncommon mutation, N = 399			P1
	N = 402	Single mutation	Complex mutation	P2	
Age				0.072	0.138
>= 65	179 (44.5)	111 (56.1)	94 (46.8)		
< 65	223 (55.5)	87 (43.9)	107 (53.2)		
Sex				0.471	0.613
Male	162 (40.3)	72 (36.4)	81 (40.3)		
Female	240 (59.7)	126 (63.6)	120 (59.7)		
ECOG PS				0.527	0.857
0~1	327 (81.3)	157 (79.3)	165 (82.1)		
2~4	75 (18.7)	41 (20.7)	36 (17.9)		
Smoking status				0.04	0.005
Never	332 (82.6)	156 (78.8)	140 (69.7)		
Ever	70 (17.4)	42 (21.2)	61 (30.3)		
Histology				0.542	0.277
Adenocarcinoma	384 (95.5)	184 (92.9)	190 (94.5)		
Non-adenocarcinoma	18 (4.5)	14 (7.1)	11 (5.5)		
Stage*				0.74	NA
I ~ IIIA	0 (0.0)	21 (10.6)	19 (9.5)		
IIIB ~ IVB	402 (100.0)	176 (88.9)	182 (90.5)		

P1: comparison between NSCLC patients with common and uncommon EGFR mutations.

P2: comparison between NSCLC patients with single and complex uncommon EGFR mutations.

*Excluding 1 patient with missing data on tumor stage

ECOG PS, Eastern Cooperative Oncology Group performance status; EGFR, epidermal growth factor receptor; NA, not applicable; NSCLC, non-small cell lung cancer.

**Table 2:** Logistic regression analysis for association factors of *EGFR* mutation status.

Variable	Univariate analysis			Multivariate analysis		
	OR	95% CI	*p* value	OR	95% CI	*p* value
Age						
>= 65	0.807	0.611–1.065	0.13	–	–	–
Sex						
Female	1.085	0.817–1.442	0.572	–	–	–
ECOG PS						
2~4	1.043	0.732–1.485	0.817	–	–	–
Smoking status						
Ever-smokers	1.65	1.175–2.329	0.004	2.37	1.545–3.668	< 0.001
Histology						
Adenocarcinoma	0.701	0.371–1.300	0.264	–	–	–

CI, confidence interval; ECOG PS, Eastern Cooperative Oncology Group performance status; EGFR, epidermal growth factor receptor; OR, odds ratios.

Of all the patients with uncommon *EGFR* mutations, 198 had one *EGFR* mutation and 201 had complex *EGFR* mutations. The clinical features and results of univariate analysis of these two populations are summarized in **Table 1**. The percentage of ever-smokers among patients with complex *EGFR* mutations was significantly greater than that among patients with single uncommon *EGFR* mutations (30.3% vs. 21.2%, *p* = 0.040).

### Smoking status and sex in patients with uncommon EGFR mutation

Since we had observed that the percentage of ever-smokers was higher in patients with uncommon *EGFR* mutations than in those with common *EGFR* mutations, as a next step, we examined the pattern of uncommon *EGFR* mutations according to smoking status and sex. The baseline characteristics between never- and ever-smokers were similar except sex (**Table 3**). Of the 198 patients with one uncommon *EGFR* mutation, 86 had *EGFR* exon 20 insertions and 112 had other single uncommon *EGFR* mutations. Of the 201 patients with complex *EGFR* mutations, 79 had *de novo* T790M mutations, 70 had complex common mutations, and 52 had complex uncommon *EGFR* mutations. The associations of smoking status and sex with uncommon *EGFR* mutations are shown in Table 4. Differences in the frequency of various types of uncommon *EGFR* mutations were only seen in the distinct smoking status but not in sex (*p* = 0.017 and 0.803, respectively). To elucidate the interaction between sex and smoking behaviors, we evaluated smoking status and observed that the proportion of ever-smokers was higher in males than in females, both in patients with common and uncommon *EGFR* mutations (41.4% vs. 1.3%, 62.7% vs. 2.8%; both *p* < 0.001).

**Table 3:** Baseline characteristics of never and ever smokers harboring uncommon *EGFR* mutations.

Characteristic, N (%)	Never-smoker	Ever-smoker	*p* value
	N = 296	N = 103	
Age			1.000
>= 65	148	52	
< 65	148	51	
Sex			< 0.001
Male	57	96	
Female	239	7	
ECOG PS			0.563
0~1	241	81	
2~4	55	22	
Histology			0.639
Adenocarcinoma	276	98	
Non-adenocarcinoma	20	5	
Stage*			0.706
I ~ IIIA	31	9	
IIIB ~ IVB	264	94	
missing	1	0	

*Excluding 1 patient with missing data on tumor stage.

ECOG PS, Eastern Cooperative Oncology Group performance status; EGFR, epidermal growth factor receptor.

**Figure 2:**
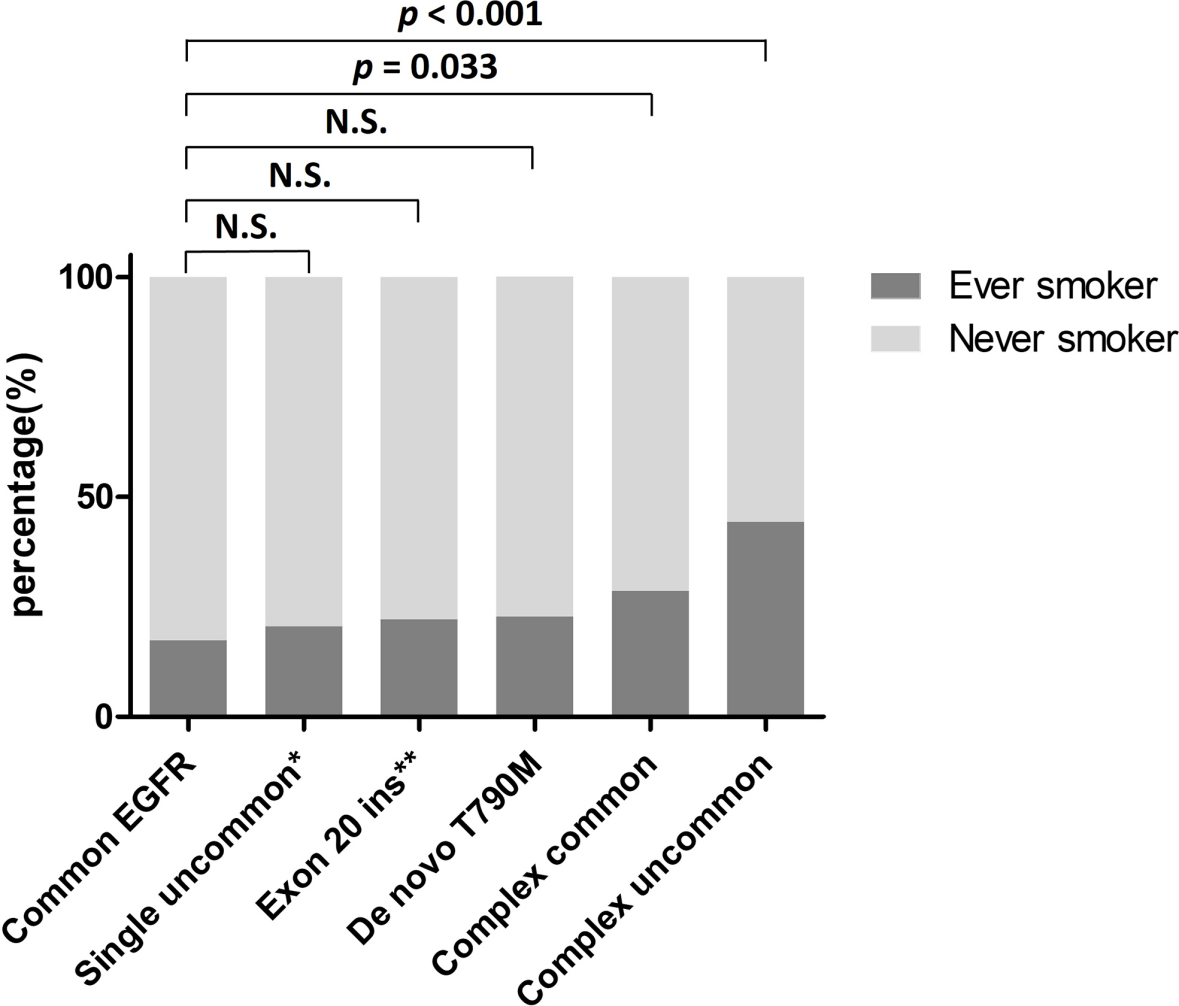
Percentage of ever-smokers in NSCLC patients harboring various *EGFR* mutations. *Excluding exon 20 insertion. **Including single exon 20 insertion only. *EGFR*, epidermal growth factor receptor; Exon 20 ins, exon 20 insertion; N.S., not significant; NSCLC, non-small cell lung cancer.

Among the 399 patients with uncommon *EGFR* mutations, ever-smokers had a significantly higher frequency of complex uncommon *EGFR* mutations than never-smokers (22.3% vs. 9.8%, *p* = 0.001). As for the remainders having exon 20 insertion, other single uncommon *EGFR* mutations, *de novo* T790M, and complex common *EGFR* mutations, the frequency was similar between ever- and never-smokers (**Table 4**). The smoking rates of patients with each uncommon *EGFR* mutation type are shown in **Figure 2**. Compared with the common *EGFR* mutation group, the percentage of ever-smokers was significantly higher in the complex common and uncommon mutation groups (*p* = 0.033 and < 0.001, respectively). In contrast, there were no significant differences in the various uncommon *EGFR* mutation types between the sexes (**Table 4**).

**Table 4:** Frequency of uncommon *EGFR* mutation types in NSCLC patients with different smoking status and sex.

Smoking status	Never-smoker	Ever-smoker	P1 value	P2 value
	N = 296	N = 103		
Uncommon *EGFR* mutations, n (%)				0.017
Single exon 20 insertion	67 (22.6)	19 (18.4)	0.373	
Single uncommon mutation except 20ins	89 (30.1)	23 (22.3)	0.132	
*De novo* T790M mutation	61 (20.6)	18 (17.5)	0.492	
Complex common mutation	50 (16.9)	20 (19.4)	0.562	
Complex uncommon mutation	29 (9.8)	23 (22.3)	0.001	
Sex	Female	Male	P1 value	P2 value
	N = 246	N = 153		
Uncommon *EGFR* mutations, n (%)				0.803
Single exon 20 insertion	52 (21.1)	34 (22.2)	0.798	
Single uncommon mutation except 20ins	74 (30.1)	38 (24.8)	0.257	
*De novo* T790M mutation	49 (19.9)	30 (19.6)	0.94	
Complex common mutation	43 (17.5)	27 (17.6)	0.966	
Complex uncommon mutation	28 (11.4)	24 (15.7)	0.214	

P1: comparison between different smoking status and sex within each uncommon EGFR mutation type.

P2: comparison between different smoking status and sex within all uncommon EGFR mutation types.

EGFR, epidermal growth factor receptor; NSCLC, non-small cell lung cancer.

### Association of smoking status with specific uncommon EGFR genotypes

To study the association of smoking behavior with specific genotypes of uncommon *EGFR* mutations, we analyzed the smoking status of patients with G719X, L861Q, and S768I. We also included patients with R776H mutations in the analysis because we noticed a high smoking rate in this population. Among all the patients with uncommon *EGFR* mutations, 96 had G719X (including 13 G719X/L861Q, 14 G719X/S768I, and 1 G719X/R776H co-mutation), 62 had L861Q (including 13 G719X/L861Q co-mutations), 25 had S768I (including 14 G719X/S768I co-mutations), and 11 had R776H (all were complex mutations). None of the patients had complex common *EGFR* mutations, with either G719X or L861Q. The smoking status of patients with G719X, L861Q, S768I, and R776H mutations are shown in **Table 3**. In patients with G719X and L861Q mutations, ever-smokers had single G719X or L861Q less frequently than never-smokers (35.3% vs. 61.3%, *p* = 0.019; 50% vs. 81.3%, *p* = 0.034, respectively). In patients with S768I mutations, ever-smokers had more G719X/S768I co-mutations (88.9% vs. 37.5%, *p* = 0.033) than never-smokers. The frequency of R776H was similar between never- and ever-smokers.

## Discussion

Several studies have identified smoking as a negative predictive marker for *EGFR* mutation status in patients with lung adenocarcinoma or NSCLC (20, 21, 28, 29). Most of these studies focused on common *EGFR* mutations. Few investigations have explored the influence of smoking on the prevalence of uncommon *EGFR* mutations, and the number of cases in these studies was limited. To the best of our knowledge, this is the largest study to explore the association between smoking status and uncommon *EGFR* mutation prevalence. In the present study, we demonstrate a comprehensive *EGFR* mutation spectrum of 3,155 patients by assessing a multi-institutional medical record database over a 10-year period. The percentage of ever-smokers was higher in the 399 patients carrying uncommon mutations than in the 402 patients carrying common mutations. A logistic regression analysis demonstrated the independent association of smoking history with uncommon *EGFR* mutation status. The frequency of complex uncommon mutations in ever-smokers was higher than that in never-smokers. Our observations suggest that smoking status is a positive predictor of specific *EGFR* mutations.

Tobacco smoke is a mixture of chemicals that contain many carcinogens (30). These carcinogens may induce DNA damage, leading to an increased burden of somatic mutations, thereby enhancing the chance of driver mutation development during tumorigenesis (22). A previous study has shown that increased somatic mutation burdens associated with smoking, including base substitutions (point mutations), small insertions and deletions (indels), and copy number changes, contribute to different extents in different cancers, but almost all contribute to lung cancer (22). Lung cancer is characterized by molecular heterogeneity. Another study demonstrated that smokers with NSCLC histology carry a 10-fold higher mutation frequency and possess a more distinct mutation spectrum than nonsmokers (31). Smoking can also trigger apolipoprotein B mRNA-editing enzyme catalytic polypeptide-like (APOBEC) activity, a major putative enzymatic source of mutation in cancers (32). One study reported that smokers with lung adenocarcinoma carried more APOBEC-associated genomic alterations compared to non-smokers (22). Furthermore, a recently proteogenomic study investigating wild-type and mutant-*EGFR* lung cancer in Taiwan revealed that APOBEC mutagenesis potentially contributed to the early onset of non-smoking lung adenocarcinoma in females (33). The study also found that high APOBEC signature was associated with younger females harboring wild-type *EGFR* and smokers with *EGFR* mutations other than L858R and Ex19del tended to have low APOBEC signature (33). In our study, we found that the frequency of complex uncommon *EGFR* mutations was significantly higher in ever-smokers than in never-smokers (**Table 4**). Compared with the common *EGFR* mutation group, the percentage of ever-smokers was significantly higher in the complex common and uncommon mutation groups (**Figure 2**). Tumor mutation burden increased by smoking may partially explain these observations. However, whether APOBEC mutagenesis contributes to the development of complex uncommon *EGFR* mutations needs to be clarified.

Although several lines of evidence have verified the negative association between smoking status and the prevalence of common *EGFR* mutations, only limited investigations have confirmed the relationship between smoking behavior and uncommon *EGFR* mutation frequency. A European study by Lohinai et al. (34) reported that rare *EGFR* mutations were significantly correlated with smoking status. In their report, 23 of 33 patients with rare mutations and 14 of 16 patients with complex uncommon mutations were smokers. Two other European studies reported similar results from cohorts of 14 and 23 patients carrying rare mutations (35, 36). However, three Asian studies showed a higher percentage of smokers in patients with uncommon *EGFR* mutations than in those with common mutations without statistical significance, even though the number of cases was higher than that in the aforementioned European studies (13, 37, 38). The Asian ethnicity is also associated with a higher prevalence of *EGFR* mutations (18). The discrepancy between eastern and western data implies that smoking may play a crucial role in uncommon *EGFR* mutagenesis. In our analysis, we enrolled a relatively large population and observed a significant difference in smoking rates between patients with common and uncommon *EGFR* mutations (**Table 1**). We further found that, compared to never-smokers, ever-smokers had a significantly divergent uncommon *EGFR* mutation spectrum (**Table 4**) and identified that patients carrying complex uncommon *EGFR* mutations had the highest smoking rate among all subgroups (**Figure 2**). Our results validate the positive relationship between smoking status and uncommon *EGFR* mutations, and provide strong evidence that smoking contributes to the development of complex *EGFR* mutations.

In addition to exon 20 insertion and *de novo* T790M mutation, G719X, L861Q, and S768I are the most frequently detected uncommon *EGFR* mutations. In the subgroup of patients harboring G719X or L861Q, we observed that never-smokers had a significantly higher frequency of single uncommon mutations than ever-smokers. Since no complex common mutation with G719X or L861Q was detected, a positive correlation between smoking status and complex uncommon mutations in the subgroup with G719X or L861Q can be implied. In patients harboring S768I, the only complex uncommon mutation was G719X/S768I, and consistently, ever-smokers had a significantly higher frequency of G719X/S768I co-mutation than never-smokers. In other words, G719X/L861Q and G719X/S768I co-mutations were the most frequent complex uncommon mutations in patients carrying these three mutations, and ever-smokers had a significantly higher frequency of these two co-mutations than never-smokers (*p* = 0.004). G719X is a mutation in exon 18. Previous surveys of exon 18 have shown that these mutations are associated with current or former smokers (39, 40). E709X and exon 18 deletions are also exon 18 mutations. Our investigation identified that 7 out of 21 patients with E709X and 1 out of 3 patients with exon 18 were ever-smokers. These findings are consistent with previous studies. Furthermore, in patients with L861Q and S768I, we first disclosed the detailed mutation spectra and their relationship with smoking status. In patients with exon 20 insertion and *de novo* T790M mutations, a low percentage of ever-smokers was observed, similar to previous reports (39, 41). In particular, we identified a subgroup of patients carrying exon 20 R776H/C mutations. All of these were co-mutations, and L858R/R776H was the most common. The smoking rate was high since six of the 12 patients were ever-smokers. A report (42) showed that R776H/C is associated with a never-smoking history, which is different from our observations. Further studies are required to confirm this relationship. Taken together, our results imply that smoking may be important in the tumorigenesis of NSCLC harboring specific uncommon *EGFR* genotypes such as G719X, L861Q, and S768I co-mutations.

Female sex has also been reported to be an important factor associated with *EGFR* mutations (19). An international prospective trial demonstrated that female sex had a statistically significant association with a higher *EGFR* mutation rate in univariate analysis, but had no association when data were stratified by smoking status (18). Gender differences in tobacco smoking were also investigated. Generally, adult males smoked more cigarettes than adult females, especially in Asian countries (43, 44). Indeed, the smoking rate was significantly higher in males than females in both the common and uncommon *EGFR* mutation cohorts in our analysis (*p* < 0.001; data not illustrated). However, the divergences in the frequency of various types of uncommon *EGFR* mutations were only seen in distinct smoking statuses but not in sex (**Table 5**). Another Japanese study revealed that *EGFR* mutations were correlated with light smoking status and adenocarcinoma histology but not sex (45). The authors proposed that a higher percentage of adenocarcinoma in females may be the reason for their predominance in the *EGFR* mutation rate. It needs to be further clarified whether sex is a determinant for *EGFR* mutation prevalence.

**Table 5:** Frequency of specific genotypes of uncommon *EGFR* mutation in never- and ever-smokers.

Genotype	Never-smoker, n (%)	Ever-smoker, n (%)	*p* value
G719X (N = 96)			
Single G719X	38 (61.3)	12 (35.3)	0.019
G719X/S768I	6 (9.7)	8 (23.5)	0.077
G719X/L861Q	7 (11.3)	6 (17.6)	0.534
G719X/E709X	5 (8.1)	4 (11.8)	0.716
G719X/T790M	2 (3.2)	2 (5.9)	0.613
Complex G719X, others*	4 (6.5)	2 (5.9)	1.000
L861Q (N = 62)			
Single L861Q	39 (81.3)	7 (50.0)	0.034
G719X/L861Q	7 (14.6)	6 (42.9)	0.056
L861Q/T790M	1 (2.1)	0 (0.0)	1.000
Complex L861Q, others*	1 (2.1)	1 (7.1)	0.404
S768I (N = 25)			
Single S768I	3 (18.8)	1 (11.1)	1.000
G719X/S768I	6 (37.5)	8 (88.9)	0.033
L858R/S768I	6 (37.5)	0 (0.0)	0.057
Ex19del/S768I	1 (6.3)	0 (0.0)	1.000
R776H (N = 11)			
L858R/R776H	3 (50.0)	5 (100.0)	0.182
Ex19del/R776H	1 (16.7)	0 (0.0)	1.000
L858R/T790M/R776H	1 (16.7)	0 (0.0)	1.000
G719X/R776H	1 (16.7)	0 (0.0)	1.000

*None with L858R or Ex19del mutations.

EGFR, epidermal growth factor receptor; Ex19del, exon 19 deletion.

The current study has several limitations. The first is its retrospective nature. Although this analysis was assessed through a multi-institutional database, potential biases, such as selection bias, could not be avoided. The second limitation was the underestimation of uncommon *EGFR* mutations. In our study, not all *EGFR* mutation detection methods used were PCR-direct sequencing. Mutant type-specific methods covered the majority of *EGFR* mutation genotypes; however, some uncommon *EGFR* mutations could not be detected. Third, no history of secondhand smoking was recorded in the medical database. Similar to first-hand smoking, second-hand smoking may have a great impact on *EGFR* mutagenesis and influence the frequency of *EGFR* mutations in never-smokers. Fourth, this study did not show the actual prevalence of uncommon *EGFR* mutations in ever- and never-smokers. We only focused on the 399 patients carrying uncommon *EGFR* mutations but did not collect data on smoking status or other epidemiological data of the entire population of 5,608 people. The comparison between ever- and never-smokers was restricted to uncommon *EGFR* mutations.

## Conclusion

*EGFR* mutations are heterogeneous genetic alterations. NSCLC harboring common *EGFR* mutations may be distinct from the ones harboring uncommon mutations. Unlike the negative association between smoking and common *EGFR* mutations, our results demonstrate a positive correlation between smoking and uncommon *EGFR* mutations. Ever-smokers had complex uncommon *EGFR* mutations more frequently than never-smokers, such as G719X, L861Q, and S768I co-mutations. Our study suggests that smoking contributes to the development of complex *EGFR* mutations.

## Data availability statement

The original contributions presented in the study are included in the article/supplementary materials. Further inquiries can be directed to the corresponding author.

## Ethics statement

The studies involving human participants were reviewed and approved by Institutional Review Board of Chang Gung Memorial Hospital (No. 202200840B0). Written informed consent for participation was not required for this study in accordance with the national legislation and the institutional requirements.

## Author contributions

H-WK, T-YY, and S-CC initiated the study concept. H-WK, T-YY, and C-CW designed the study. H-WK, S-SS, C-LW, C-YL, C-HK, Y-CL, L-FL, C-TY, and C-CW contributed to the data acquisition. C-WW validated the pathology data. H-WK and C-TC performed the data analysis. H-WK and C-CW interpreted the data. H-WK contributed to the funding acquisition and drafted the manuscript. C-TY and C-CW supervised the study. All authors contributed to the article and approved the submitted version.

## Funding

This study was supported by the grant MOST 108-2314-B-182A-131 from the Ministry of Science and Technology, Taiwan, and grants CMRPG3J1951 and CMRPG5L0171 from Linkou Chang Gung Memorial Hospital.

## Acknowledgments

We thank the research assistant, Ms. Shin-Yu Huang, for this study. We also thank Editage, a division of Cactus Communications, for editorial assistance.

## Conflict of interest

The authors declare that the research was conducted in the absence of any commercial or financial relationships that could be construed as a potential conflict of interest.

## Publisher’s note

All claims expressed in this article are solely those of the authors and do not necessarily represent those of their affiliated organizations, or those of the publisher, the editors and the reviewers. Any product that may be evaluated in this article, or claim that may be made by its manufacturer, is not guaranteed or endorsed by the publisher.
